# Supplementary material for: Spatial Regulation of Lysosomal Vesicle Acidification Along the Axon via mRAVE-Dependent v-ATPase Assembly
Source: bioRxiv. 2025 Dec 23:2025.12.22.696043. Preprint. [Version 1] doi: 10.64898/2025.12.22.696043 (PMC12776041; doi:10.64898/2025.12.22.696043)
Supplement: Supplement 1 [file NIHPP2025.12.22.696043v1-supplement-1.pdf]

Figure S1

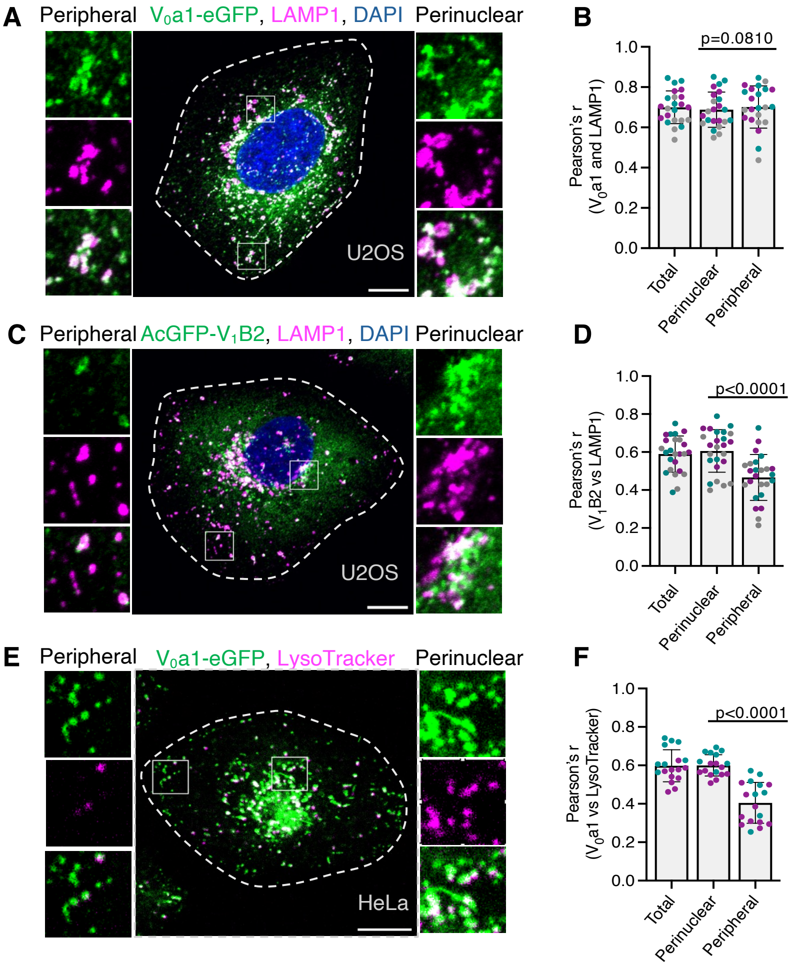

Figure S1. Co-localization of v-ATPase subunits V_0a1 and V_1B2 with lysosomal markers in HeLa and U2OS cells. (A,C) Immunofluorescence microscopy showing co-localization of stably expressed V_0a1–eGFP (green) (A) or AcGFP–V_1B2 (green) (C) with endogenous LAMP1 (magenta) in U2OS cells. Nuclei were stained with DAPI (blue). Magnified views of the boxed areas in the peripheral and perinuclear regions are shown at left and right, respectively. **(B,D)** Quantification of the co-localization between LAMP1 and V_0a1–eGFP (B) or AcGFP–V_1B2 (D) in total, perinuclear, and peripheral regions, expressed as Pearson's correlation coefficients (Pearson's r), from experiments such as those shown in panels A and C (*n*=23-25 cells from three independent experiments). **(E)** Single frame live-cell images of HeLa cells showing the co-localization of stably expressed V_0a1–eGFP (green) with LysoTracker (magenta). Magnified views of the boxed areas in the peripheral and perinuclear regions are shown at left and right, respectively**. (F)** Quantification of the co-localization between LysoTracker and V_0a1–eGFP in total, perinuclear, and peripheral regions, expressed as Pearson's r, from experiments such as those shown in panel E (*n*=18-19 cells from two independent experiments). All quantitative data are represented as the mean ± SD. Statistical significance was assessed using the Friedman test with Dunn's multiple comparisons test. Actual *P* values are indicated in the figure. Scale bars: 10 µm.

Figure S2

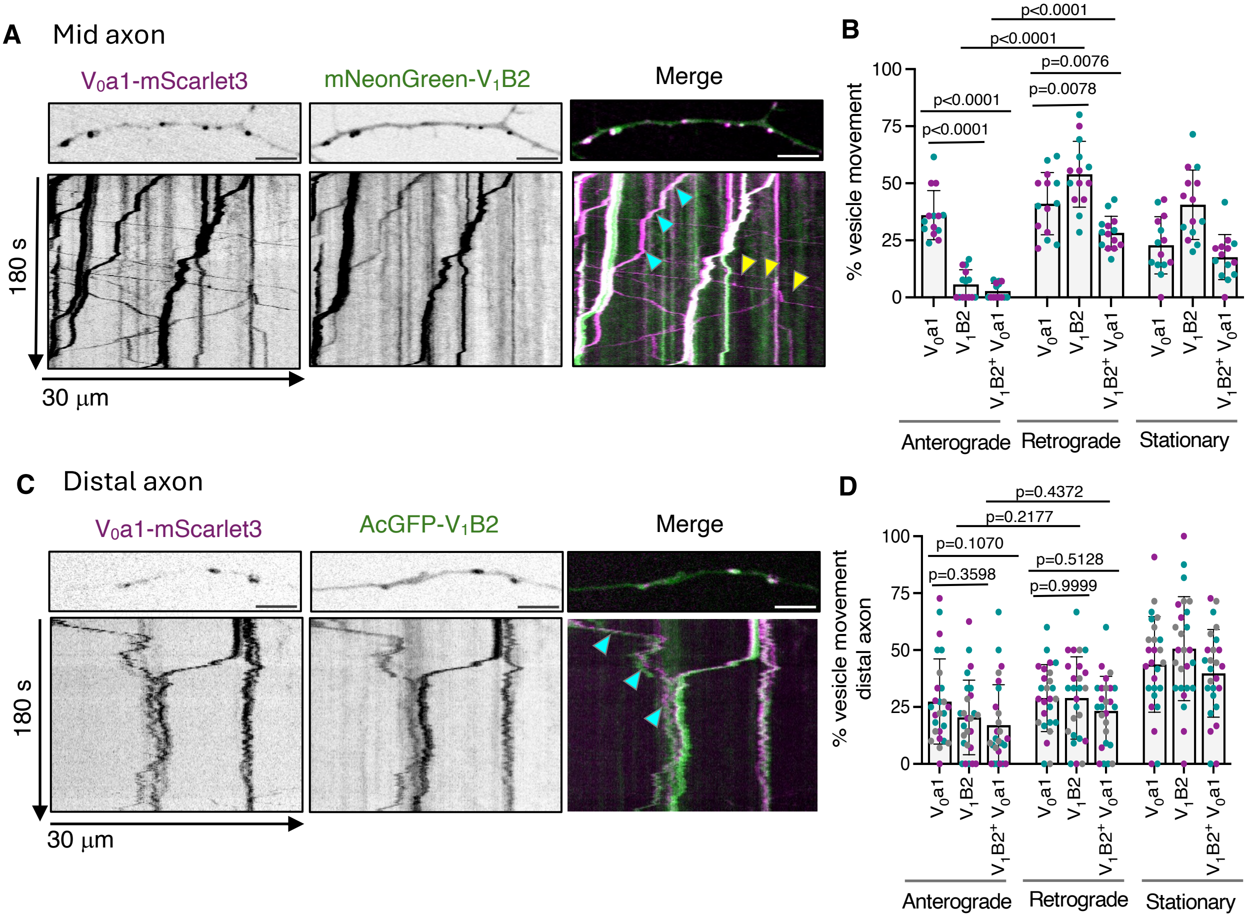

Figure S2. Transport dynamics of vesicles containing V_0a1 and V_1B2 subunits in rat hippocampal neurons. (A) Single frames (top) and corresponding kymographs (bottom) of 30-μm axonal segments located approximately 50 μm from the soma of DIV7 rat hippocampal neurons co-expressing V_0a1–mScarlet3 and mNeonGreen–V_1B2 imaged live for 180 s. **(B)** Quantification of the proportion of anterograde, retrograde, and stationary vesicles in axons from neurons co-expressing V_0a1–mScarlet3 and mNeonGreen–V_1B2 from experiments such as that shown in panel A (n=14 neurons from ≥4 cultures prepared from two rats). Cyan arrowheads indicate V_1-V_0-positive vesicles and yellow arrows indicate V_0-only-positive vesicles. **(C)** Single frames (top) and corresponding kymographs (bottom) of 30-μm distal axonal segments, located approximately 20 μm from the axon tip of DIV7 rat hippocampal neurons co-expressing V_0a1–mScarlet3 and AcGFP–V_1B2, imaged live for 180 s. Cyan arrowheads indicate V_1-V_0-positive vesicles. **(D)** Quantification of the proportion of anterograde, retrograde, and stationary vesicles in axons from neurons co-expressing V_0a1–mScarlet3 and AcGFP–V_1B2 from experiments such as that shown in panel C (n=26 neurons from ≥4 cultures prepared from three rats). Statistical significance was calculated using two-way ANOVA with Tukey's multiple comparisons test for panel F. Actual P values are indicated in the figure. Scale bars: 5 μm.

Movies

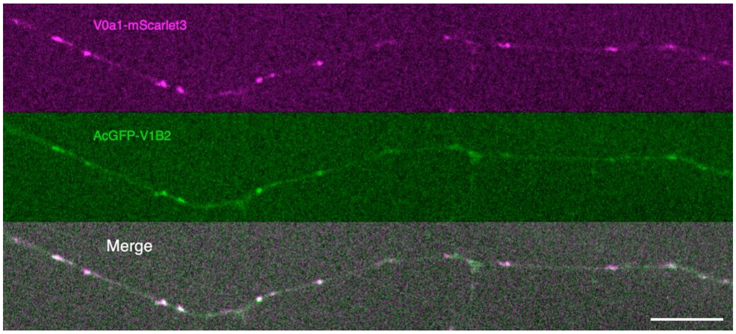

Video S1. Video showing a 100-µm long section from the axon shaft of a DIV7 rat hippocampal neuron co-expressing V_0-mScarlet3 (magenta) and AcGFP-V1B2 (green) and imaged by live-cell spinning-disk fluorescence microscopy. The time-series was recorded for 180 s with 1 s between frames. Notice the bidirectional movement of V_0a1, retrograde movement of V_1B2, and merged vesicles (white). Scale bar: 10 µm

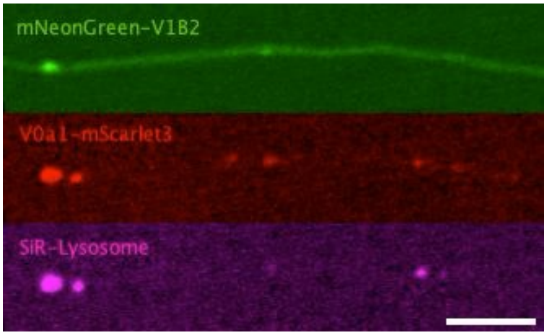

Video S2. Video showing a 30-µm long section from the axon shaft of a DIV7 rat hippocampal neuron co-expressing V_0a1-mScarlet3 (red) and mNeon-GreenV_1B2 (green), stained with SiR-lysosome (magenta) and imaged by live-cell spinning-disk fluorescence microscopy. The time-series was recorded for 60 s at 1 s intervals. Notice the co-movement of SiR-lysosome with V_0a1 and V_1B2 vesicles. Scale bar: 5 µm
